# The Prognostic Value of Immune Factors in the Tumor Microenvironment of Penile Squamous Cell Carcinoma

**DOI:** 10.3389/fimmu.2018.01253

**Published:** 2018-06-11

**Authors:** Sarah Rosanne Ottenhof, Rosa Sanne Djajadiningrat, Helene Hoegsbro Thygesen, Pamela Josephine Jakobs, Katarzyna Jóźwiak, Anne Marijne Heeren, Jeroen de Jong, Joyce Sanders, Simon Horenblas, Ekaterina Straschimirova Jordanova

**Affiliations:** ^1^Department of Urology, Netherlands Cancer Institute, Amsterdam, Netherlands; ^2^Department of Urology, HagaZiekenhuis, Hague, Netherlands; ^3^Department of Biometrics, Netherlands Cancer Institute, Amsterdam, Netherlands; ^4^Statistics, Department of Conservation, Hamilton, New Zealand; ^5^Department of Epidemiology and Biostatistics, Netherlands Cancer Institute, Amsterdam, Netherlands; ^6^Center for Gynecologic Oncology Amsterdam (CGOA), VU University Medical Center, Amsterdam, Netherlands; ^7^Department of Pathology, Netherlands Cancer Institute, Amsterdam, Netherlands

**Keywords:** B7-H1, HPV, immune escape, microenvironment, penile cancer, programmed death ligand-1, squamous cell carcinoma, T-cells

## Abstract

The host’s immune system plays a pivotal role in many tumor types, including squamous cell carcinomas (SCCs). We aim to identify immunological prognosticators for lymph node metastases (LNM) and disease-specific survival (DSS) in penile SCC. For this retrospective observational cohort study, penile SCC patients (*n* = 213) treated in the Netherlands Cancer Institute, were selected if sufficient formalin-fixed, paraffin-embedded tumor material was available. Analysis included previously described high-risk human papilloma virus (hrHPV) status, immunohistochemical scores for classical and non-classical human leukocyte antigen (HLA) class I, programmed death ligand-1 (PD-L1) expression, and novel data on tumor-infiltrating macrophages and cytotoxic an regulatory T-cells. Clinicopathological characteristics and extended follow-up were also included. Regression analyses investigated relationships of the immune parameters with LNM and DSS. In the total cohort, diffuse PD-L1 tumor-cell expression, CD163^+^ macrophage infiltration, non-classical HLA class I upregulation, and low stromal CD8^+^ T-cell infiltration were all associated with LNM. In the multivariable model, only tumor PD-L1 expression remained a significant predictor for LNM (odds ratio (OR) 2.8, *p* = 0.05). hrHPV negativity and diffuse PD-L1 tumor-cell expression were significantly associated with poor DSS and remained so upon correction for clinical parameters [hazard ratio (HR) 9.7, *p* < 0.01 and HR 2.8, *p* = 0.03]. The only immune factor with different expression in HPV^+^ and HPV^−^ tumors was PD-L1, with higher PD-L1 expression in the latter (*p* = 0.03). In the HPV^−^ cohort (*n* = 158), LNM were associated with diffuse PD-L1 tumor-cell expression, high intratumoral CD163^+^ macrophage infiltration, and low number of stromal CD8^+^ T-cells. The first two parameters were also linked to DSS. In the multivariable regression model, diffuse PD-L1 expression remained significantly unfavorable for DSS (HR 5.0, *p* < 0.01). These results emphasize the complexity of the tumor microenvironment in penile cancer and point toward several possible immunotherapy targets. Here described immune factors can aid risk-stratification and should be evaluated in clinical immunotherapy studies to ultimately lead to patient tailored treatment.

## Introduction

Penile squamous cell carcinoma (SCC) is a rare disease with an incidence of less than 1/100,000 in Western countries (1, 2). The prognosis for early stage penile cancer patients is good (5-year survival without lymphogenic spread is 96%) but worsens gradually with presence of lymph node metastases (LNM) (2, 3). Surgery is the mainstay of penile cancer treatment, for both primary tumors and LNM. Only in advanced stages (e.g., pelvic lymph node involvement or irresectable disease) multimodal treatment is necessary, mostly in the form of neoadjuvant chemotherapy or adjuvant radiation (4).

In 20–50% of the patients, penile SCC is induced by a persistent infection with high-risk human papilloma virus (hrHPV) (5, 6). Diagnosis, treatment, and follow-up are the same for hrHPV-negative (hrHPV^−^) and hrHPV-positive (hrHPV^+^) tumors (4). Nevertheless, patients with hrHPV^+^ tumors have a better disease-specific survival (DSS) than patients with hrHPV^−^ tumors (5-year DSS of 96 vs. 82% respectively) (7).

The difference in patient outcomes between hrHPV^+^ and non-virally induced penile cancer may be partially explained by different immune escape mechanisms (8–14). Surely, immunosuppressive and immunostimulating factors in the tumor microenvironment (TME) co-determine the course of disease in many different cancers, but relatively little is known about penile SCC (15, 16).

For example, in head and neck squamous cell carcinomas (HNSCCs) higher levels of tumor-infiltrating immune cells in hrHPV^+^ tumors are indicated as pivotal role players in a better response to standard therapy in comparison to hrHPV^−^ tumors (17–19). This concerns high levels of intratumoral CD8^+^ and CD3^+^ T-lymphocytes but also antigen presenting cells such as myeloid dendritic cells (18–21). CD8^+^ cytotoxic T-cells are capable of immediate tumor-cell killing and therewith are the effectors of anti-tumor response (21). Regulatory T-cells (Tregs) are well known for their detrimental effect on the immune response (10, 12, 22). However, associations of Tregs with clinical outcome remain controversial. High numbers of FoxP3^+^ Tregs were associated with early stage disease and better overall survival in HNSCC, but with adverse patient outcome in colorectal cancer and non-small-cell lung carcinoma (18, 23–25). Cytotoxic and Treg subpopulations have both been described as prognostic factors separately, as well as the ratio between the two (15, 19, 20, 26). An increased CD8/FoxP3-ratio at diagnosis has been associated with responsiveness to immunotherapy in renal cancer and melanoma (15, 27–29). Tumor-infiltrating macrophages (TIM) are usually macrophages with an immunosuppressive M2-phenotype (30–32). These macrophages are marked by CD163 and are associated with T-cell response suppression, migration, and treatment evasion (30, 31). High CD163^+^ macrophage infiltration was associated with high disease stage and LNM in hrHPV^+^ cervical cancer, and with poor survival in oral SCC (32, 33).

In penile cancer, various immune escape mechanisms in the TME have been studied (partly by our group) (8–14). In a multivariable analysis by Vassallo et al., presence of FoxP3-positive lymphocytes (presumably Tregs) was associated with poor disease free survival (10). In addition, a decreased CD8/FoxP3-ratio was associated with tumor progression during follow-up (12). Human leukocyte antigen (HLA) class I was assessed with immunohistochemical (IHC) staining on a tissue microarray (TMA). A prognostic role was only found for HLA-A expression that was associated with decreased overall survival (9). No differences in HLA expression were observed between HPV^−^ and HPV^+^ tumors. Programmed death ligand-1 (PD-L1) expression was assessed in multiple studies, using different antibodies and techniques (10–14). HPV^−^ penile cancer cells are more often PD-L1^+^. Tumor-cell PD-L1-expression was associated with worse DSS and LNM, especially a diffuse expression of PD-L1 throughout the tumor fields (11, 13, 14).

To compare the prognostic value of all these parameters, and to determine which factors have the strongest associations with patient outcomes, different factors from the TME should be evaluated in an integrative analysis. The aim of this study was to gain insight in the TME, and to identify possible associations between TME factors and LNM/DSS in patients with penile cancer.

In this retrospective observational cohort study, we investigated previously determined factors (HPV status, classical and non-classical HLA class I, and PD-L1 expression) in combination with novel data on tumor-infiltrating cytotoxic T-cells, Tregs, and M2-polarized macrophages (7, 9, 11).

## Materials and Methods

### Study Population and Tissue Samples

Between 2001 and 2009, 487 consecutive patients were diagnosed with penile SCC in the Netherlands Cancer Institute, Amsterdam. All were considered for inclusion, according to the following criteria. Exclusion criteria were non-invasive carcinoma, neoadjuvant non-surgical treatment, no tumor tissue available in our institutional biobank (mostly because of surgical removal elsewhere or treatment with laser ablation). Inclusion criterion was that sufficient archived tissue needed to be available in our institutional biobank. Sufficient archived formalin-fixed, paraffin-embedded (FFPE) material was available from 216 patients. All were staged and surgically treated in a standardized way (34). Clinical follow-up data were updated. Patients were usually clinically followed for 5 years, after that, patient status was sometimes available through municipal administration. This study was carried out with approval of the institutional medical ethical committee that considered this study not falling within the scope of the act of research involving human subjects, it was also approved by the translational research board of our institute.

Evaluation of the IHC stainings on 5 µm sections was performed by two researchers (Rosa Sanne Djajadiningrat and Ekaterina Straschimirova Jordanova or Sarah Rosanne Ottenhof and Ekaterina Straschimirova Jordanova) and an experienced uropathologist (Jeroen de Jong). Three patients were excluded because a majority of the parameters could not be analyzed (e.g., no invasive tumor present in sample).

### hrHPV-Typing

For protocols of hrHPV-typing, classical HLA, non-classical HLA, and PD-L1 IHC analyses, we refer to our previous reports (7, 9, 11). In short, hrHPV status was determined on 212 tissue samples using GP5^+^/6^+^ PCR enzyme-immunoassay for 14 different HPV types (7).

### Immunohistochemistry

A TMA of 168 samples was immunohistochemically analyzed for HLA class I expression with the following antibodies: HCA2 (HLA-A), HC10 (HLA-B/C; both provided by Prof. Neefjes of our institute), anti-beta-2-microglobulin (β2m; DAKO, Denmark), MEM-E/02 (HLA-E; Bio-Rad, USA), and 4H84 (HLA-G; from BD Pharmingen, USA) (9). PD-L1 was determined on 213 whole-mount sections using the E1L3N clone (Cell Signaling, USA) (11).

Whole-mount sections from 213 FFPE tissue blocks were immunohistochemically stained for CD8 (C8/144B, DAKO, Denmark), FoxP3 (236A/E7, AbCam, England), and CD163 (MRQ-26, Cell Marque, Rocklin, USA) using the Ventana protocol and autostainer with heat induced antigen retrieval. Details of different IHC stainings are summarized in Table S1 in Supplementary Material.

### Immunofluorescent Double Staining

Twelve randomly selected cases (six hrHPV^−^ and six hrHPV^+^ tumors) were double-stained with primary antibodies CD163 (10D6, NCL-CD163, Novocastra, Germany) and CD68 (514H12, MCA1815, Bio-Rad, UK). Secondary antibodies from Life Technologies, USA were used for detection. The slides were analyzed manually using a fully motorized digital imaging fluorescence microscope (Axiovert-200M, Germany). More details of these stainings can be found in Table S1 in Supplementary Material.

### Scoring Methods

Human leukocyte antigen-A, HLA-B/C, and β2m expression were scored in a semiquantitative way with the quality control system proposed by Ruiter et al. using intensity and percentage, resulting in three categories: negative, weak or positive (9, 35). A combined score of HLA-A, HLA-B/C, and β2m grouped tumors into categories of classical HLA class I expression: normal expression (all three positive), complete downregulation (negative β2m or negative HLA-A and HLA-B/C), and partial downregulation (other combinations). Although HLA-A was significant in previous multivariable analysis of this cohort, the total score of classical HLA was used for analysis because it had stronger associations with updated variables (comparative data not shown) (9). HLA-E and HLA-G were scored as absent/upregulated, and a combined score resulted in two groups: tumors into normal expression of non-classical HLA class I (both negative) and upregulation (one or both upregulated).

Only membranous staining of PD-L1 was scored. Percentage of positive cells was noted, cut-off for PD-L1 positivity of tumors was ≥1% of tumor cells (11, 12, 36, 37). For PD-L1^+^ tumors, the tumor expression pattern was scored as diffuse (throughout the tumor fields) or margin (predominantly at the tumor-stroma margin) (11). Immune cells in stroma were scored binary (negative or positive). PD-L1-positive TIM were identified by size, shape, end position (large, round, with dendrites, and in tumor fields) and were scored as present or absent (11).

For CD8^+^ and FoxP3^+^ T-cell infiltration analysis, in each sample three peripheral and three central tumor focus fields were randomly selected in Aperio ImageScope (Leica Biosystems, Solms, Germany) and magnified by 20×. Each image (focus field) contained stroma and tumor fields. The number of positive pixels was determined with the semi-automatic computer program Image-J (NIH, Bethesda, MD, USA; http://rsb.info.nih.gov/ij/). Images were deconvoluted with a plug-in to the color red. By setting a threshold (at 180 for every image), the positive pixels were separated from the negative pixels. For every image tumor fields were digitally selected. The size of the total image area, tumor area and stromal area in pixels was noted, together with the number of positive pixels in these areas. The stromal values were calculated by subtracting the tumor area from the whole image area. In each tumor slide, the average number of positive pixels in the six focus fields was used for both CD8 and FoxP3 in tumor area and stromal area. T-cell ratios were calculated by dividing the CD8^+^ pixels by FoxP3^+^ pixels.

Semiquantitative analysis of CD163 in tumor and stroma determined low/high infiltration of CD163^+^ cells. The 12 immunofluorescently stained samples (CD163/CD68) were qualitatively analyzed.

### Statistical Analysis

**Figure 1:**
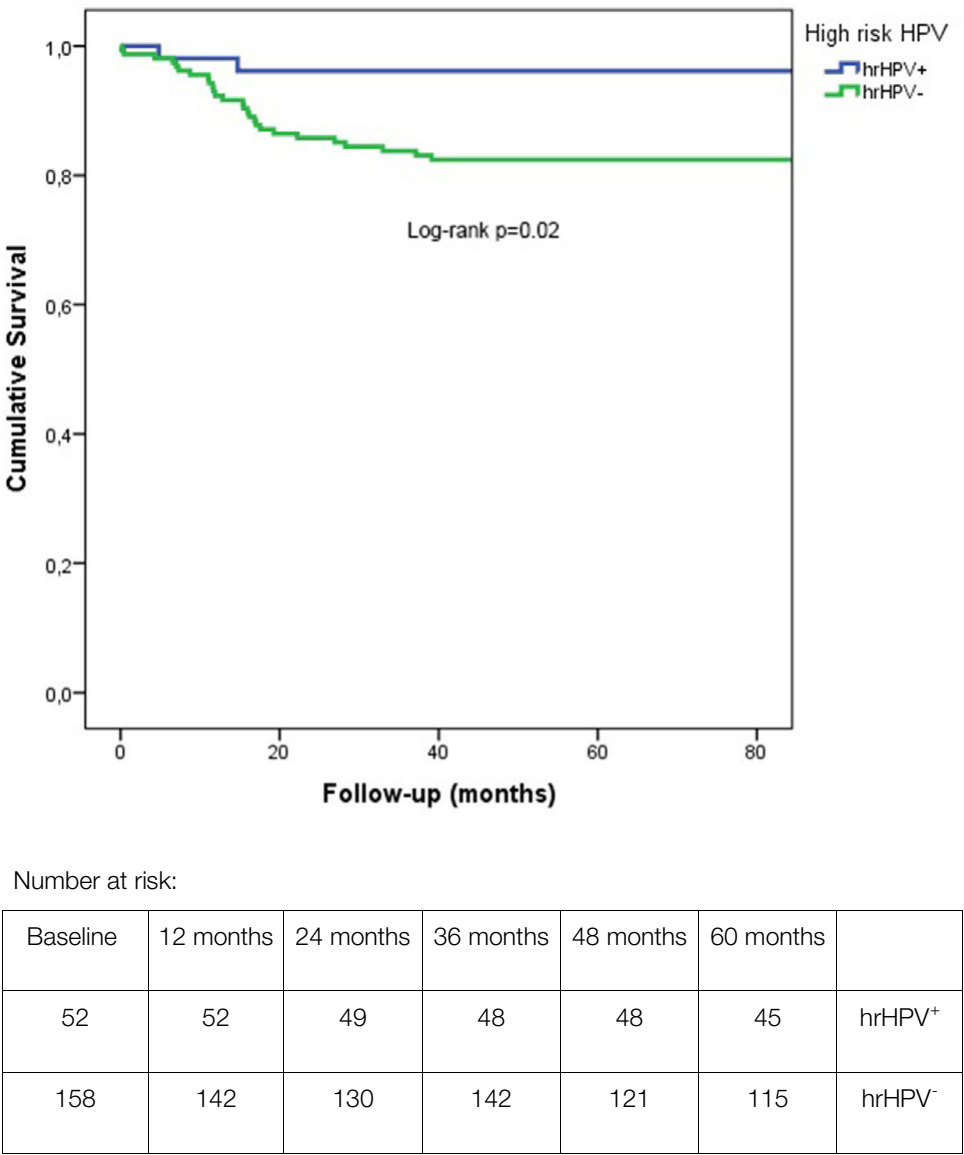
Kaplan–Meier survival plots with log-rank test analysis of high-risk HPV-positive and -negative penile cancer cases.

High-risk human papilloma virus subgroups were compared with respect to clinicopathological, tumor and stroma characteristics using chi-square test, Fishers’ exact test, and *t*-tests for independent samples. Also, Kaplan–Meier estimated survival curves were plotted for HPV groups (Figure 1). Normality was assessed with Kolmogorov–Smirnov for all continuous parameters. T-cell parameters were transformed to log-scale to meet normality assumption when comparing means (*t*-test). Pixel counts of CD8 and FoxP3 were divided by 100,000 for statistical analyses so that hazard ratios (HRs) and odds ratios (ORs) represent a substantial change. A constant integer (of 1) was added to stromal CD8 and stroma FoxP3 to prevent division by zero when calculating T-cell ratios. A logistic regression was used to model odds of LNM, and a Cox regression to model DSS from date of diagnosis to death from penile cancer or last follow-up/death from other cause. Characteristics that were significant or nearly significant in univariable models, were considered for final multivariable models found with a backward stepwise selection approach with models comparison using likelihood-ratio tests and *p* > 0.10 as covariate exclusion criterion. All analyses were done using SPSS^®^ version 22 (IBM, Armonk, NY, USA) in collaboration with experienced statisticians (Helene Hoegsbro Thygesen and Katarzyna Jóźwiak).

## Results

### Clinicopathological Characteristics

In this cohort (*n* = 213), 68 patients (31.9%) had LNM, and 87 patients (40.8%) died during follow-up; 29 patients (13.6%) died of penile cancer (on average after 14.7 months). Median overall follow-up was 100.7 months (IQR 69.4–119.7). Mean DSS was 166.8 months (median not reached).

Age was normally distributed. Tumor size was not. T-cell parameters (intratumoral and stroma CD8 and FoxP3, and T-cell ratios) were normally distributed after log-scale transformation. Clinicopathological characteristics are summarized in Table 1. When comparing the hrHPV subgroups with respect to these characteristics, we observed a significant difference only in differentiation grade (*p* < 0.01) and death by penile cancer (*p* = 0.02). Most well differentiated tumors were hrHPV^−^ (70 vs. 9 in hrHPV^+^). Despite this, DSS was better in hrHPV^+^ patients in comparison to hrHPV^−^ patients, with 2 and 27 penile cancer related deaths, respectively (log-rank *p* = 0.02; Figure 1) at mean follow-up of 169.5 vs. 160.5 months. Among hrHPV^+^ tumors, HPV16 was the predominant type 79% (41/52) (7).

**Table 1:** Clinicopathological characteristics.

Variable	hrHPV^**−**^, *N* = 158 (%)	hrHPV^**+**^,*N* = 52 (%)	Total, *N* = 213 (%)^a^	*p*-Value^b^
**Age** median (IQR)	67.6 (58.2–74.6)	63.6 (54.4–71.6)	65.9 (57.3–74.4)	0.38
**pT stage**				0.17
pT1	42 (26.6)	19 (36.5)	61 (28.6)	
pT2	99 (62.7)	28 (53.8)	130 (61.0)	
pT3	11 (7.0)	5 (9.6)	16 (7.5)	
pT4	6 (3.8)	–	6 (2.8)	
**Tumor size** median (IQR)	3.0 (2.0–4.1)	2.5 (1.5–3.9)	3.0 (2.0–4.0)	0.09
**Histological subtype**			0.08^c^
SCC NOS	137 (87.3)	43 (82.7)	180 (84.5)	
Papillary	8 (5.1)	1 (1.9)	9 (4.2)	
Verrucous	5 (3.2)	–	5 (2.3)	
Warty	2 (1.3)	3 (5.8)	5 (2.3)	
Basaloid	1 (0.6)	4 (7.7)	5 (2.3)	
Mixed SCC-basaloid	1 (0.6)	1 (1.9)	2 (0.9)	
Sarcomatoid	1 (0.6)	–	1 (0.5)	
Cuniculatum	1 (0.6)	–	1 (0.5)	
Pseudohyperplastic	1 (0.6)	–	1 (0.5)	
Missing	1 (0.6)	–	4 (1.9)	
**Grade of differentiation**				**<0.01**
Well (grade 1)	70 (44.3)	9 (17.3)	80 (37.6)	
Intermediate (grade 2)	62 (39.2)	31 (59.6)	94 (44.1)	
Poor (grade 3)	26 (16.5)	12 (23.1)	38 (17.8)	
Missing	–	–	1 (0.5)	
**pN stage**				0.84
pN0	107 (67.7)	36 (69.2)	145 (68.1)	
pN+	51 (32.3)	16 (30.8)	68 (31.9)	
**Extranodal growth**				0.12^d^
Present	19 (12.0)	3 (5.8)	22 (10.3)	
Absent	28 (17.7)	13 (25)	42 (19.7)	
No LNM	107 (67.7)	36 (69.2)	145 (68.1)	
Missing	4 (2.5)	–	4 (1.9)	
**Death by penile cancer**				**0.02**
No	131 (82.9)	50 (96.2)	184	
Yes	27 (17.1)	2 (3.8)	29	

*IQR, interquartile range (25th–75th percentile); SCC, squamous cell carcinoma; NOS, not otherwise specified; HPV, human papilloma virus; LNM, lymph node metastases*.

*^a^Including three cases with unknown hrHPV status*.

*^b^Excluding missing cases. Comparing the two hrHPV subgroups. Independent sample t-test for continuous variables, chi-square, or Fishers exact test for categorical variables*.

*^c^Divided in SCC NOS, unfavorable subtypes (basaloid, mixed basaloid-warty, and sarcomatoid) and favorable subtypes (papillary, verrucous, warty, cuniculatum, and pseudohyperplastic)*.

*^d^Excluding patients with no LNM/unknown lymph node status*.

*Bold numbers are statistically significant*.

### Classical and Non-Classical HLA Expression and PD-L1 Expression Patterns

**Figure 2:**
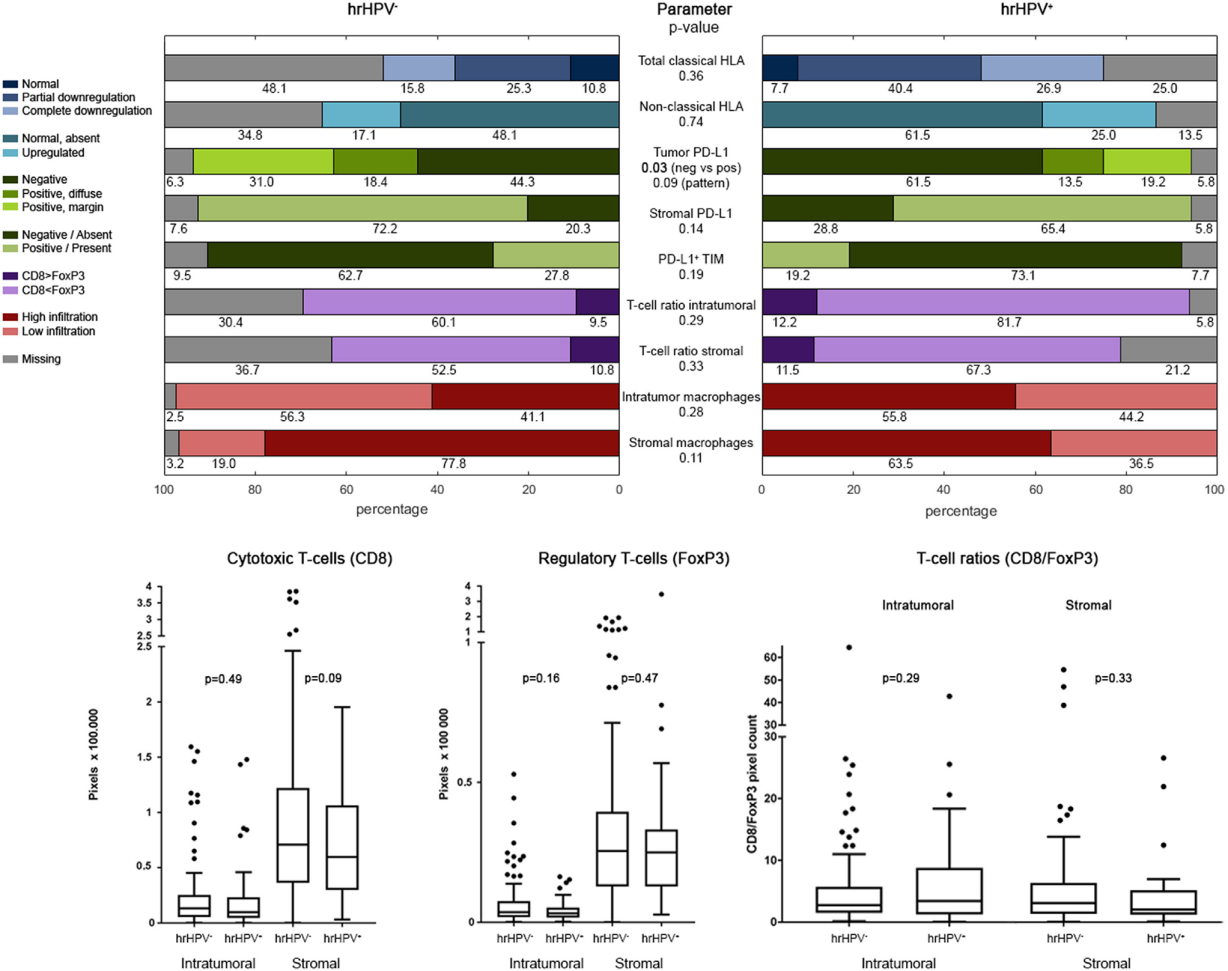
Tumor-microenvironmental characteristics. Plots of immune parameters in hrHPV^−^ and hrHPV^+^ samples; categorical variables in bar plots, continuous variables in box plots. *p*-Values of testing comparable distribution in hrHPV groups. Table S2 in Supplementary Material presents the same data. Abbreviations: HLA, human leukocyte antigen; PD-L1, Programmed death ligand-1; TIM, tumor-infiltrating macrophages; hrHPV, high-risk human papilloma virus. Expression of PD-L1 on tumor cells was compared in two ways: negative vs. positive (neg vs. pos) and negative vs. positive at margin vs. diffusely positive (pattern).

Immune characteristics are summarized in Figure 2 and Table S2 in Supplementary Material. Aberrant classical and non-classical HLA expression was equally distributed among hrHPV^−^ subgroups. Interestingly, hrHPV^−^ tumors were significantly more often PD-L1^+^ (49.4 vs. 32.7% of hrHPV^+^; *p* = 0.03). Also, there was a trend toward hrHPV^−^ tumors having relatively more of both PD-L1 expression patterns compared with hrHPV^+^ tumors (*p* = 0.09) (11).

### Tumor-Infiltrating Cytotoxic T-Cells and Tregs

**Figure 3:**
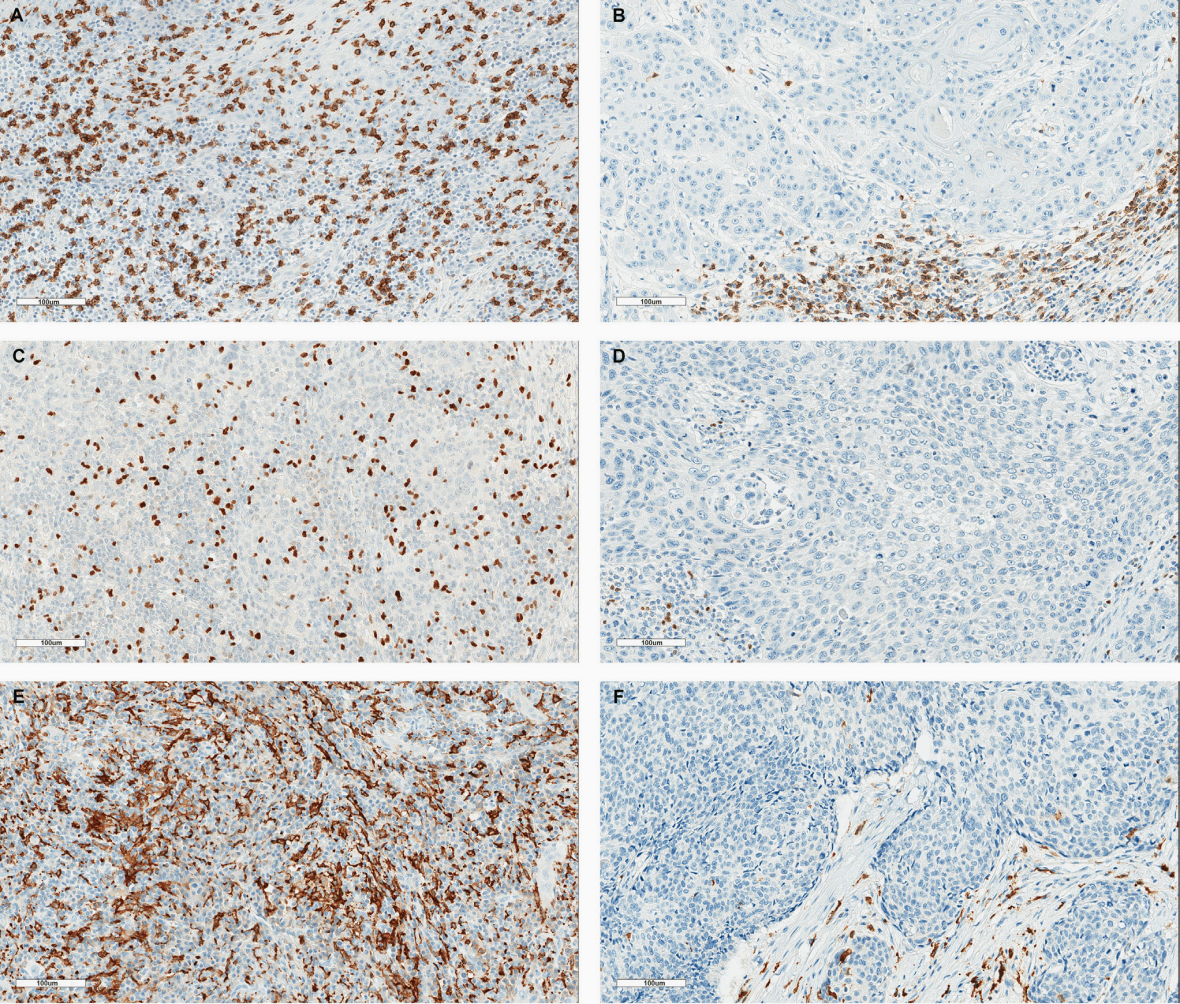
Examples of representative stainings for high and low infiltration of CD8^+^ T cells **(A,B)**, FoxP3^+^ regulatory T-cells **(C,D)**, and CD163^+^ macrophages **(E,F)**. Scale bars: 100 µm.

The presence of CD8^+^ T-cells and FoxP3^+^ Tregs was determined by standard IHC staining. Representative examples of CD8 and Foxp3 presence are depicted in Figures 3A–D. Interestingly, CD8 and FoxP3 pixel counts were much higher in stromal areas than in tumor areas, in both hrHPV^−^ and hrHPV^+^ tumors (Figure 2). No differences in T-cell numbers or CD8/FoxP3-ratio were found between hrHPV^+^ and hrHPV^−^ tumors (Figure 2; Table S2 in Supplementary Material).

### Tumor-Infiltrating Macrophages

Representative examples of CD163 IHC stainings are depicted in Figures 3E,F. No significant differences in CD163^+^ macrophage intratumoral or stromal infiltration were observed between hrHPV^−^ and hrHPV^+^ samples.

**Figure 4:**
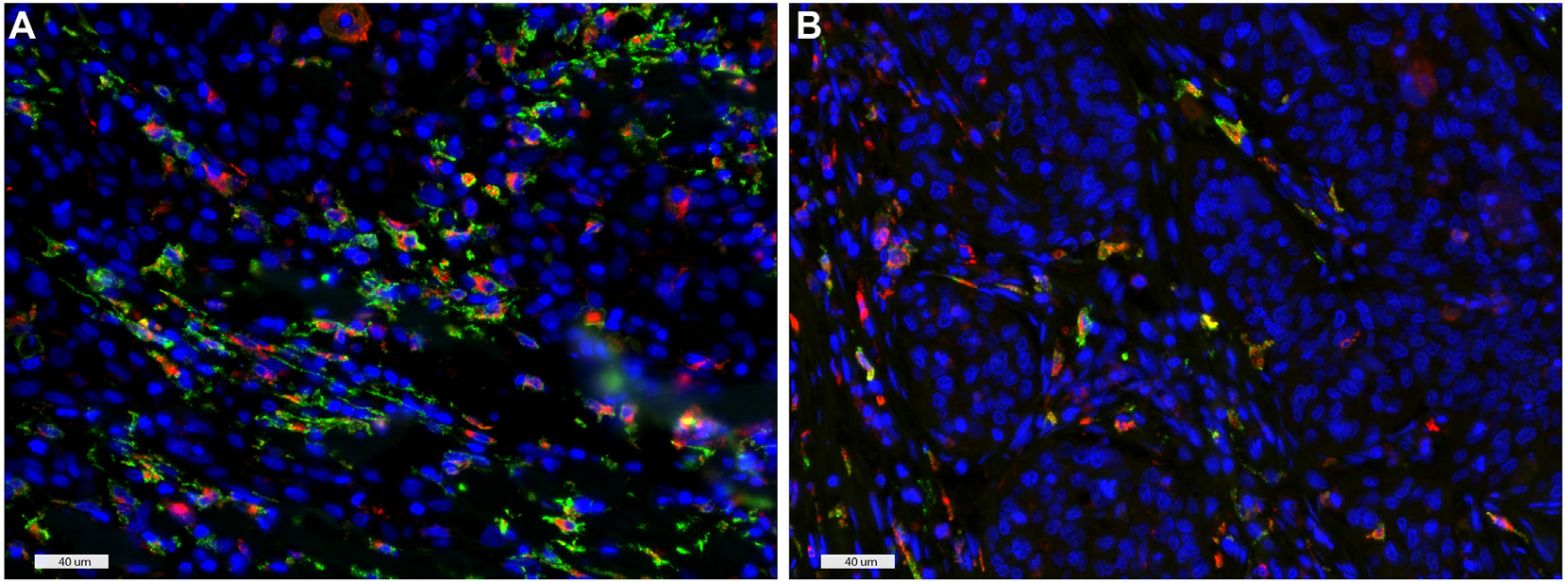
CD163 and CD68 double staining of an hrHPV^−^ **(A)** and hrHPV^+^ **(B)** case, indicative of M2 macrophage polarization. Colors: green, CD163; red, CD68; and blue, DAPI. Scale bars: 40 µm.

In addition, to determine the subtype of macrophages infiltrating penile tumors, a fluorescent double staining of CD163 and CD68 was performed (Figures 4A,B) and the majority of cells were found to be CD68^+^CD163^+^ both intratumoral and in stromal areas, indicative of M2-polarization of virtually all macrophages in these tumors.

### Univariable Analyses

#### Associations Between TME Factors and LNM

Results from the univariable analysis are presented in Table 2. With clinicopathological parameters and updated follow-up of patients, results resembled our previous reports (7, 9, 11). Tumor PD-L1 expression was significantly associated with LNM; diffusely PD-L1-positive tumors had higher odds of LNM in comparison to tumors with marginal PD-L1 expression only [OR 4.16, *p* < 0.01] and to tumors with combined negative/margin PD-L1 expression (OR 3.28, *p* < 0.01). Presence of PD-L1^+^ TIM was associated with higher chance of LNM but not on a level of conventional statistical significance (OR 1.91, *p* > 0.05). The presence of high numbers of intratumoral CD163^+^ M2 macrophages was significantly associated with higher LNM incidence (OR 2.45, *p* < 0.01).

**Table 2:** Univariable analysis.

Variable	Contrast	Lymph node metastasis (LNM)		Disease-specific survival (DSS)	
		OR [CI]	*p*-Value	HR [CI]	*p*-Value
**Tumor-microenvironmental parameters**					
High-risk HPV	Negative vs. positive	1.07 [0.55–2.11]	0.84	4.82 [1.15–20.27]	**0.03**
PD-L1 pattern	Negative vs. margin	1.44 [0.68–3.03]	0.34	1.28 [0.44–3.68]	0.65
	Diffuse vs. margin	4.16 [1.71–10.17]	**<0.01**	4.35 [1.53–12.34]	**<0.01**
	Diffuse vs. negative/margin	3.28 [1.58–6.84]	**<0.01**	3.70 [1.75–7.82]	**<0.01**
PD-L1 stroma	Positive vs. negative	0.78 [0.39–1.55]	0.48	1.38 [0.52–3.63]	0.52
PD-L1 TIM	Present vs. absent	1.91 [0.99–3.70]	>0.05	1.74 [0.79–3.83]	0.17
Classical HLA class I	Complete vs. partial downregulation	1.26 [0.45–3.58]	0.18	0.12 [0.02–0.96]	**<0.05**
	Normal expression vs. partial downregulation	0.69 [0.22–2.19]	0.66	0.45 [0.10–2.00]	0.29
Non-classical HLA class I	Upregulated vs. normal	2.28 [1.08–4.81]	**0.02**	0.53 [0.15–1.84]	0.32
CD8 intratumoral	Per 100,000 pixels	1.32 [0.50–3.50]	0.58	0.83 [0.21–3.31]	0.79
CD8 stromal	Per 100,000 pixels	0.60 [0.37–0.98]	**0.04**	0.84 [0.49–1.44]	0.52
FoxP3 intratumoral	Per 100,000 pixels	24.74 [0.40–1,532.10]	0.13	36.39 [0.92–1,433.75]	0.06
FoxP3 stromal	Per 100,000 pixels	0.54 [0.18–1.62]	0.27	0.61 [0.14–2.57]	0.50
T-cell ratio intratumoral	Continuous	1.01 [0.97–1.05]	0.71	0.96 [0.88–1.05]	0.39
T-cell ratio stromal	Continuous	0.98 [0.92–1.04]	0.42	1.00 [0.93–1.06]	0.93
CD163 intratumoral	High vs. low infiltration	2.45 [1.35–4.43]	**<0.01**	2.10 [0.99–4.44]	>0.05
CD163 stromal	High vs. low infiltration	1.75 [0.85–3.62]	0.13	1.99 [0.69–5.74]	0.20

**Clinicopathological parameters**					
Age	Per year	1.00 [0.98–1.02]	0.95	1.01 [0.98–1.04]	0.40
Tumor size	Per cm	1.11 [0.97–1.26]	0.13	1.21 [1.07–1.37]	
pT stage	pT2 vs. pT1a/b	2.33 [1.10–4.91]	**0.03**	1.32 [0.48–3.67]	0.59
	pT3–4 vs. pT1a/b	6.57 [2.25–19.17]	**<0.01**	7.19 [2.46–21.07]	**<0.01**
Grade of differentiation	Intermediate vs. good differentiation	8.01 [3.16–20.27]	**<0.01**	2.11 [0.74–5.98]	0.16
	Poor vs. good differentiation	21.14 [7.32–61.11]	**<0.01**	5.99 [2.11–17.01]	**<0.01**
LVI	Present vs. absent	4.65 [2.11–10.23]	**<0.01**	3.21 [1.45–7.10]	**<0.01**
Exophytic growth	Present vs. absent	0.62 [0.32–1.18]	0.14	0.65 [0.28–1.53]	0.33
Lymph node status	pN+ vs. pN0	–	–	38.51 [9.15–162.16]	**<0.01**
Extranodal growth^a^	Present vs. absent	–	–	2.11 [0.93–4.78]	0.08

*OR, odds ratio; HR, hazard ratio; LVI, lymphovascular invasion; HPV, human papilloma virus; HLA, human leukocyte antigen; PD-L1, programmed death ligand-1; TIM, tumor-infiltrating macrophages*.

*^a^Excluding cases with no LNM*.

*Bold numbers are statistically significant*.

Aberrant classical HLA class I expression patterns (combined score of HLA-A, HLA-B/C, and β2m) did not show significant associations with LNM. Interestingly, upregulation of non-classical HLA class I molecules (combined score of HLA-E and HLA-G) was associated with a higher odds of LNM compared with normal expression (OR 2.28, *p* = 0.02).

The only T-cell infiltration parameter showing significant association with LNM, was increased CD8^+^ T-cell infiltration rate in tumor-associated stroma (OR 0.60, *p* = 0.04) albeit with a confidence interval (95% CI) almost including 1: 0.37–0.98.

#### Associations Between TME Factors and DSS

High-risk human papilloma virus negativity was associated with worse survival (HR 4.82, *p* = 0.03), and complete downregulation of classical HLA class I with better survival than partial downregulation (HR 0.12, *p* < 0.05, note questionable 95% CI of 0.02–0.96) (Table 2). A diffuse PD-L1 tumor expression pattern was associated with higher risk of disease-specific death than marginal PD-L1 expression (HR 4.35, *p* < 0.01), and negative/margin PD-L1 expression (HR 3.70, *p* < 0.01).

Although we saw some evidence of associations of DSS with intratumoral Tregs (HR 36.39, *p* = 0.06) and high intratumoral CD163^+^ M2-macrophage infiltration (HR 2.10, *p* ≥ 0.05), these associations were not significant.

### Multivariable Analysis

Classical and non-classical HLA were non-significant in the multivariable models (data not shown). These variables limited the number of included cases in the multivariable models because of a relatively high number of missing values, and therefore they were excluded from the final models to increase the sample size.

In the multivariable analysis (Table 3), diffuse PD-L1 expression was the only immunological factor that remained significantly associated with LNM, although the lower limit of the confidence interval was just above 1 (OR 2.81, 95% CI [1.01–7.81], *p* < 0.05). hrHPV negativity and diffuse PD-L1 expression were immune factors predicting poor survival in the multivariable model (OR 9.73, *p* < 0.01, and OR 2.78, *p* = 0.03, respectively).

**Table 3:** Multivariable backward regression analysis.

Lymph node metastasis			

Variable	Contrast	OR [CI]	*p*-Value
Tumor PD-L1	Diffuse vs. negative/margin	2.81 [1.01–7.81]	**0.05**
PD-L1^+^ TIM	Present vs. absent	–	–
CD8 stromal	Per 100,000 pixels increase	0.54 [0.27–1.05]	0.07
CD163 intratumoral	High vs. low infiltration	–	–
LVI	Present vs. absent	3.18 [1.08–9.35]	**0.04**
Grade of differentiation	Intermediate vs. good differentiation	6.76 [2.11–21.63]	**<0.01**
	Poor vs. good differentiation	12.07 [3.19–45.70]	**<0.01**
pT stage	pT2 vs. pT1a/b	–	–
	pT3–4 vs. pT1a/b	–	–

**Disease-specific survival**			

**Variable**	**Contrast**	**HR [CI]**	*****p***-Value**

High-risk HPV	Absent vs. present	9.73 [2.12–44.72]	**<0.01**
Tumor PD-L1	Diffuse vs. negative/margin	2.78 [1.10–6.98]	**0.03**
FoxP3 intratumoral	Per 1,000 pixels increase	–	–
CD163 intratumoral	High vs. low infiltration	–	–
Tumor size	Per cm	1.31 [1.11–1.53]	**<0.01**
Lymph node status	pN+ vs. pN0	63.21 [12.36–323.23]	**<0.01**
Grade of differentiation	Intermediate vs. good differentiation	0.30 [0.09–1.06]	0.06
	Poor vs. good differentiation	0.87 [0.26–2.90]	0.81

*OR, odds ratio; CI, 95% confidence interval for OR/HR; PD-L1, programmed death ligand-1; TIM, tumor-infiltrating macrophages LVI, lymphovascular invasion; HR, hazard ratio; HPV, human papilloma virus; –, excluded during regression analysis*.

*Bold numbers are statistically significant*.

### Subgroup Analyses

hrHPV^+^ and hrHPV^−^ penile cancer can be seen as two different tumor entities, and patients with hrHPV^−^ tumors have a higher risk of dying from this disease (7). Also, various histological subtypes of SCC have a distinct better or poorer prognosis (38). Therefore, analyses were repeated in the hrHPV^−^ subgroup, and the subgroup with usual histological subtype SCC (Tables 4 and 5).

**Table 4:** Univariable subgroup analysis.

Univariable subgroup analysis					

hrHPV^**−**^ subgroup(*N* = 158)		Lymph node metastasis (LNM)		Disease-specific survival (DSS)	
			
Variable	Contrast	OR [CI]	*p*-Value	HR [CI]	*p*-Value
PD-L1 pattern	Negative vs. margin	1.67 [0.71–3.96]	0.24	1.86 [0.58–5.93]	0.30
	Diffuse vs. margin	4.18 [1.53–11.43]	**<0.01**	6.19 [2.00–19.22]	**<0.01**
	Diffuse vs. negative/margin	3.04 [1.32–7.01]	**<0.01**	4.15 [1.92–8.98]	**<0.01**
PD-L1 stroma	Positive vs. negative	0.98 [0.42–2.27]	0.95	1.42 [0.49–4.15]	0.52
PD-L1 TIM	Present vs. absent	1.52 [0.72–3.25]	0.28	1.46 [0.64–3.33]	0.37
Total classical HLA class I	Partial downregulation vs. normal expression	2.17 [0.60–7.85]	0.24	0.13 [0.02–1.01]	0.05
	Complete downregulations vs. normal expression	1.26 [0.31–5.23]	0.75	0.37 [0.08–1.66]	0.19
Total non-classical HLA class I	Upregulated vs. normal	1.84 [0.75–4.55]	0.15	0.59 [0.17–2.07]	0.41
CD8 intratumoral	Per 100,000 pixels	0.83 [0.24–2.85]	0.77	0.80 [0.18–3.62]	0.77
CD8 stromal	Per 100,000 pixels	0.45 [0.23–0.88]	**0.02**	0.80 [0.45–1.43]	0.46
FoxP3 intratumoral	Per 100,000 pixels	18.21 [0.25–1,345.66]	0.19	22.69 [0.58–891.96]	0.10
FoxP3 stromal	Per 100,000 pixels	0.71 [0.22–2.27]	0.56	0.52 [0.12–2.35]	0.40
T-cell ratio intratumoral	Continuous	1.00 [0.95–1.05]	0.96	0.93 [0.81–1.05]	0.24
T-cell ratio stromal	Continuous	0.95 [0.86–1.04]	0.24	0.99 [0.93–1.06]	0.74
CD163 intratumoral	High vs. low infiltration	2.17 [1.10–4.30]	**0.03**	2.17 [1.10–4.30]	**0.05**
CD163 stromal	High vs. low infiltration	1.43 [0.59–3.48]	0.44	1.23 [0.59–3.48]	0.44

**Usual SCC subgroup(***N*** = 180)**		**LNM**		**DSS**	
			
**Variable**	**Contrast**	**OR [CI]**	*****p***-Value**	**HR [CI]**	*****p***-Value**

High-risk HPV	Negative vs. positive	1.17 [0.56–2.45]	0.68	4.04 [0.96–17.10]	0.06
PD-L1 pattern	Negative vs. margin	1.37 [0.62–3.04]	0.44	1.65 [0.52–5.17]	0.39
	Diffuse vs. margin	3.17 [1.23–8.18]	**0.02**	4.22 [1.32–13.45]	**0.02**
	Diffuse vs. negative/margin	2.58 [1.17–5.67]	**0.02**	3.01 [1.35–6.69]	**<0.01**
PD-L1 stroma	Positive vs. negative	0.79 [0.37–1.68]	0.54	1.12 [0.42–3.00]	0.82
PD-L1 TIM	Present vs. absent	2.10 [1.05–4.20]	**0.04**	1.70 [0.75–3.82]	0.20
Total classical HLA class I	Partial downregulation vs. normal expression	1.20 [0.41–3.52]	0.74	0.00 [0.00–0.00]	0.96
	Complete downregulations vs. normal expression	0.48 [0.13–1.75]	0.27	0.46 [0.10–2.12]	0.32
Total non-classical HLA class I	Upregulated vs. normal	2.14 [0.97–4.75]	0.06	0.59 [0.17–2.08]	0.41
CD8 intratumoral	Per 100,000 pixels	1.21 [0.38–3.80]	0.75	0.79 [0.17–3.60]	0.76
CD8 stromal	Per 100,000 pixels	0.55 [0.32–0.93]	**0.03**	0.81 [0.47–1.40]	0.45
FoxP3 intratumoral	Per 100,000 pixels	40.50 [0.53–3,085.53]	0.09	19.38 [0.34–1,114.01]	0.15
FoxP3 stromal	Per 100,000 pixels	0. 0.59 [0.20–1.74]	0.34	0.69 [0.18–2.74]	0.60
T-cell ratio intratumoral	Continuous	0.98 [0.92–1.04]	0.51	0.97 [0.88–1.06]	0.45
T-cell ratio stromal	Continuous	0.96 [0.90–1.03]	0.24	0.99 [0.93–1.06]	0.92
CD163 intratumoral	High vs. low infiltration	2.36 [1.25–4.48]	**<0.01**	2.28 [1.02–5.11]	**0.05**
CD163 stromal	High vs. low infiltration	2.03 [0.89–4.59]	0.09	1.60 [0.55–4.65]	0.39

*OR, odds ratio; HR, hazard ratio; CI, 95% confidence interval for OR/HR; LVI, lymphovascular invasion; hrHPV, high-risk human papilloma virus; HLA, human leukocyte antigen; PD-L1, Programmed death ligand-1; TIM, tumor-infiltrating macrophages; SCC, squamous cell carcinoma; –, excluded from the multivariable model during regression analysis; NA, not applicable*.

*Bold numbers are statistically significant*.

**Table 5:** Multivariable subgroup analysis.

Multivariable subgroup analysis					

Lymph node metastasis (LNM)		hrHPV^−^		Usual SCC	
			
Variables included in model	Contrast	OR [CI]	*p*-Value	OR [CI]	*p*-Value
Tumor PD-L1	Diffuse vs. negative/margin	–	–	–	–
PD-L1^+^ TIM	Present vs. absent	–	–	–	–
CD8 stromal	Per 100,000 pixels increase	0.44 [0.18–1.05]	0.06	0.38 [0.18–0.81]	**0.01**
CD163 intratumoral	High vs. low infiltration	–	–	–	–
LVI	Present vs. absent	3.91 [0.93–18.37]	0.08	–	–
Grade of differentiation	Intermediate vs. good differentiation	15.30 [3.86–60.66]	**<0.01**	6.09 [1.82–20.44]	**<0.01**
	Poor vs. good differentiation	19.34 [3.92–95.53]	**<0.01**	19.11 [4.34–84.11]	**<0.01**
pT stage	pT2 vs. pT1	–	–	1.43 [0.45–4.48]	0.55
	pT3–4 vs. pT1	–	–	10.14 [1.39–73.84]	**0.02**

**Disease-specific survival**		**hrHPV^−^**		**Usual SCC**	
			
**Variables included in model**	**Contrast**	**HR [CI]**	*****p***-Value**	**HR [CI]**	*****p***-Value**

High-risk HPV	Absent vs. present	NA	NA	6.82 [1.49–31.37]	**0.01**
Tumor PD-L1	Diffuse vs. negative/margin	5.03 [1.81–13.99]	**<0.01**	2.48 [0.91–6.80]	0.08
FoxP3 intratumoral	Per 1,000 pixels increase	183.89 [0.96–35,153.22]	>0.05	–	–
CD163 intratumoral	High vs. low infiltration	–	–	–	–
Tumor size	Per cm	1.47 [1.23–1.76]	**<0.01**	1.32 [1.12–1.55]	**<0.01**
Lymph node status	pN+ vs. pN0	82.22 [14.99–450.90]	**<0.01**	124.33 [14.51–1,065.27]	**<0.01**
Grade of differentiation	Intermediate vs. good differentiation	0.25 [0.07–0.91]	**0.04**	0.25 [0.07–0.92]	**0.04**
	Poor vs. good differentiation	0.84 [0.25–2.86]	0.78	0.71 [0.22–2.34]	0.57

*hrHPV, high-risk human papilloma virus; OR, odds ratio; CI, 95% confidence interval; PD-L1, programmed death ligand-1; TIM, tumor-infiltrating macrophages; LVI, lymphovascular invasion; HR, hazard ratio; SCC, squamous cell carcinoma; –, excluded from the multivariable model during regression analysis*.

*Bold numbers are statistically significant*.

#### hrHPV-Negative Subgroup

In univariable analysis of the hrHPV^−^ subgroup (*n* = 158), three factors were significantly associated with LNM: a diffuse PD-L1 expression pattern (OR 4.18, *p* < 0.01), high intratumoral infiltration rates of CD163^+^ macrophages (OR 2.17, *p* = 0.03), and—associated with lower risk of LNM—high stromal CD8^+^ T-cell infiltration (OR 0.45, *p* = 0.02). Diffuse PD-L1 expression pattern and high intratumoral CD163^+^ macrophage infiltration were also significantly associated with worse DSS (HR 6.19, *p* < 0.01 and HR 2.17, *p* < 0.05).

Multivariable regression analysis of the hrHPV^−^ subgroup, showed grade of differentiation as the only significant factor associated with LNM (OR 15.30 and 19.34 for grades 2 and 3 compared with grade 1, both *p* < 0.01). High stromal CD8^+^ T cell infiltration showed some evidence of negative association with LNM but was not statistically significant (OR 0.44, *p* = 0.06). PD-L1 expression pattern was eliminated during backward selection.

For DSS in the hrHPV^−^ subgroup, LNM (HR 82.22, *p* < 0.01) and diffuse PD-L1 expression pattern (OR 5.03, *p* < 0.01) remained the most important factors in the multivariable model. High FoxP3^+^ Treg infiltration rates were associated with worse DSS but did not meet statistical significance (OR 183.89, *p* ≥ 0.05).

#### Usual Histological Subtype SCC

Univariable analysis in the usual histological subtypes (*n* = 180), showed—similar to the hrHPV^−^ subgroup—significant associations with LNM for PD-L1 expression pattern (OR 3.17, *p* = 0.02) and high intratumoral CD163^+^ macrophage infiltration rates (OR 2.36, *p* < 0.01), and a negative association with LNM for high stromal CD8^+^ T-cell infiltration (OR 0.45, *p* = 0.03) (Tables 4 and 5). Unlike the hrHPV^−^ subgroup, LNM were also associated with PD-L1-expressing TIM (OR 2.10, *p* = 0.04). Poor DSS was associated with presence of diffuse PD-L1 expression pattern (HR 4.22, *p* = 0.02) and high intratumoral CD163^+^ macrophage infiltration (HR 2.28, *p* < 0.05).

After multivariable regression, the final model for LNM included grade of differentiation (similar values as hrHPV^−^), high stromal CD8 (OR 0.38, *p* = 0.01) and pT stage (OR 10.14, *p* = 0.02 for T3/T4 vs. T1). Like in the hrHPV^−^ subgroup, PD-L1 was eliminated during backward selection. For DSS, having lymph node metastases was the most important predictor of survival (HR 124.33, *p* < 0.01). The multivariable model also included hrHPV negativity (HR 6.82, *p* < 0.01) and other clinical predictors.

## Discussion

This is the largest study that reports on associations of multiple TME factors with patient outcomes adjusted for clinical predictors in penile cancer.

In the total cohort, diffuse PD-L1 tumor-cell expression, CD163^+^ macrophage infiltration, non-classical HLA class I upregulation and low stromal CD8^+^ T-cell infiltration, were all associated with LNM. In the multivariable model, only PD-L1 expression remained a significant predictor for LNM (OR 2.81, *p* = 0.05). hrHPV negativity and diffuse PD-L1 tumor-cell expression were significantly associated with poor DSS and remained so upon correction for clinical parameters (HR 9.73, *p* < 0.01 and HR 2.81, *p* = 0.03).

The strong prognostic value for hrHPV reflects two tumor entities, similar to head-and-neck SCC and vulvar SCC (39–41). One is hrHPV-mediated, more immunogenic, and associated with better prognosis (41, 42). The other is HPV-independent, induced by chronic irritation, inflammation and genetic alterations (39, 40, 43). Interestingly, the only immune factor that differed from HPV^+^ to HPV^−^ tumors was PD-L1 expression, with higher PD-L1 expression rates in the latter (*p* = 0.03). In the HPV^−^ cohort (*n* = 158), LNM were associated with diffuse PD-L1 tumor-cell expression, high intratumoral CD163^+^ macrophage infiltration and low number of stromal CD8^+^ T-cells, while only the first two parameters were associated with DSS. In the HPV^−^ subgroup multivariable regression model, diffuse PD-L1 expression remained significantly associated with poor DSS (HR 5.03, *p* < 0.01). Similar results were obtained when the cohort analysis was restricted to the usual histological subtype SCC.

The contrasting associations of diffuse PD-L1 expression with poor outcomes and PD-L1 expression at the tumor-stroma margin with more favorable outcomes can be explained by two different pathways of PD-L1 expression, identified in melanoma and gynecological SCC (44–47). The first has a genetic background (deregulated signaling pathways, transcription factors and numerical aberrations) resulting in *CD274* overexpression, and concomitant diffuse PD-L1 expression (15, 44, 46). The other is a reactive, interferon-gamma (IFNγ) induced expression at the tumor-stroma margin, explaining its favorable role (45, 47). We hypothesized that the better survival of cases with tumor-margin PD-L1 expression is explained by accumulation of activated T-cells and IFNγ release in the adjacent stroma (11). But among the PD-L1-positive tumors, stromal CD8^+^ T-cell infiltration was not associated with a marginal expression pattern (data not shown, Spearman, *p* = 0.819). The higher number of diffusely PD-L1 positive tumors in the hrHPV^−^ group of our cohort, however, fits the hypothesis of a more mutated tumor type with higher T-cell inhibition properties, partially explaining poorer survival. Deng et al. studied PD-L1 expression and tumor-infiltrating lymphocytes in penile cancer and also did functional analyses on cell lines (14). They found PD-L1 expression positively correlated with IFNγ and CD8^+^ gene expression, suggesting that indeed PD-L1 expression was induced by activated T-cells (14, 45). The proportion of hrHPV^+^ tumors in their study is presumably low (prevalence in Asia around 13%) (14). Recent studies in oropharyngeal SCC reported on a prognostic role for CD8^+^ T-cell infiltration rates and not for PD-L1 expression (17, 48). Like us, Oguejiofor et al. found higher PD-L1 expression in HPV^−^ tumors (17). However, they also investigated CD8^+^ T-cells expressing the PD-L1 receptor PD-1 and found higher proportions of CD8^+^PD-1^+^ T-cells in stroma than in tumor. Considering higher PD-L1 expression in hrHPV^−^ tumors, this suggests pronounced T-cell inhibition in this unfavorable group. In HNSCC, CD8^+^ T-cells were more frequent in HPV^+^ tumors, and also more capable of producing IFNγ (20). Another study found that not only composition but also location of suppressive factors matter; PD-L1^+^ or FoxP3^+^ cells close to CD8^+^ T-cells (within 30 µm) are associated with worse overall survival (48). We did not assess PD-1 expression, IFNγ-producing capacity or proximity of suppressive factors in our cohort, but these factors may influence the different outcomes of patients with hrHPV^+^ and hrHPV^−^ tumors.

Cocks et al. found a decreased CD8^+^ T cell/FoxP3^+^ Treg-ratio associated with tumor progression during follow-up in penile cancer patients, but no associations with overall survival or DSS (12). We also found no associations with this ratio and did not use progression during follow-up as outcome. These discrepancies can be partially explained by technical differences (they performed hot-spot analysis in TMAs). But also by factors that are not included in our analysis, such as other checkpoint molecules (e.g., CTLA-4) and PD-1 expression on T-cells.

Based on our results, can we inverse tumor escape in penile carcinomas, and how?

First, with PD-L1 as one of the most important predictors of prognosis in penile SCC, trials with PD-(L)1-checkpoint-inhibitors are warranted. Systemic treatment with these agents has been FDA-approved for various cancers, including SCCs (49). In the Netherlands Cancer Institute, we are currently planning a clinical trial with such agents in advanced penile cancer.

Second, the favorable high stromal CD8^+^ T cell and low intratumoral CD163^+^ macrophage infiltration should be notified as important mechanisms. M2-polarized macrophages play a crucial role in T-cell response suppression, angiogenesis and treatment evasion, but can be reprogrammed toward activated M1 macrophages by CD4^+^ helper T-cells (30, 31, 50). In the future, combinational immunotherapies should be applied to counter the adverse effects of the complex microenvironment in these tumors (51).

Limitations of the study include the relatively few cases with LNM and disease associated deaths in this cohort, and the substantial missing values in HLA expression due to insufficient tissue material for TMA sampling (9). Both limited the statistical analysis. Second, we did not determine PD-1 expression, distance from CD8^+^ T-cells to PD-L1 expressing tumor cells and tumor-associated macrophages, or functionality (48). Furthermore, our results ideally are externally validated.

Nevertheless, our results favor the rationale for immunotherapy for this mutilating disease. Any effectiveness of immunotherapy on primary tumor or LNM has to be revealed by future clinical studies, stratifying patients based on TME parameters, eventually leading to personalized immunotherapy. We are currently focusing on comparing the TME of primary tumors to metastatic lymph nodes.

In conclusion, in this study, we showed that the penile cancer microenvironment is highly complex and contains various targets for immunotherapy. These results can aid risk-stratification and importantly, the here described TME factors should be evaluated in future immunotherapy clinical studies to ultimately lead to patient tailored treatment.

## Data Availability Statement

Datasets are available on request.

## Author Contributions

This study was designed by SH and EJ. Scoring of samples was done by SO, RD, PJ, AH, JJ, JS, and EJ. Clinicopathological data were collected by SO and RD, parts of it were revised by JJ. AH performed CD163/CD86 double stainings. Statistical analysis was done by SO, HT, and KJ. The manuscript was drafted by SO, sections of it were written by AH and EJ. The manuscript was critically reviewed, read, and approved by all co-authors.

## Conflict of Interest Statement

The authors declare that the research was conducted in the absence of any commercial or financial relationships that could be construed as a potential conflict of interest.
