# Supplementary material for: The Prognostic Value of Immune Factors in the Tumor Microenvironment of Penile Squamous Cell Carcinoma
Source: Front Immunol. 2018 Jun 11;9:1253. doi: 10.3389/fimmu.2018.01253 (PMC6004546; doi:10.3389/fimmu.2018.01253)
Supplement: Supplementary file 1 [file table_1.PDF]

Supplementary Table 1: Immunohistochemical stainings.

Antibody	Clone	Catalog number	Manufacturer	Tissue sections & preparation	Heat induced antigen retrieval	Primary antibody incubation	Secondary antibody	Detection	Counterstain
HLA-A	HCA2	-	Provided by prof. Neefjes (NCI, The Netherlands)	Tissue microarray. Deparaffinisation and rehydration with graded ethanol to distilled water. Endogenous peroxidase activity was blocked with 0.03% H_2O_2/ MeOH for 20'.	10' boiling 0.01M citrate buffer at pH 6.0 for HLA-A, HLA-B/C, β2M, and HLA-E.<br><br>10' boiling Tris/EDTA buffer at pH 9.0	1:600 Overnight, RT	30' BrightVision (ImmunoLogic BV, The Netherlands)	10' applying a 0.05M Tris–HCl buffer containing 0.05% of 3,3'-diamino-benzidine-tetrahydrochloride and 0.0018% H_2O_2.	Mayer's haematoxylin
HLA-B/C	HC10	-				1:1000 Overnight, RT			
β2 micro-globulin	A0072	anti-β-2M	DAKO, Denmark			1:4000 Overnight, RT			
HLA-E	MEM-E/02	MCA2193	Bio-Rad, USA (formerly AbD Serotec, UK)			1:250 Overnight, RT			
HLA-G	4H84	557577	BD Pharmingen, USA			1:100 Overnight, RT			
CD8	C8/144B	M7103	DAKO / Agilent, Denmark	Whole mount sections. BenchMark Ultra autostainer (Ventana Medical Systems, USA). Paraffin sections were heated (28' 75°C) and deparaffinized with EZ prep solution (Ventana Medical Systems, USA).	32' 95°C pH8.5 Cell conditioning 1 (Ventana Medical Systems, USA)	1:100 32' 37°C	OptiView DAB Detection Kit (Ventana Medical Systems, USA)		Hematoxylin II and Blueing Reagent (Ventana Medical Systems, USA)
PD-L1	E1L3N	13684	Cell Signaling Technology, USA		64' 95°C pH8.5 Cell conditioning 1 (Ventana Medical Systems, USA)	1:200 60' RT			
FoxP3	236A/E7	vab20034	AbCam, UK			1:40 120' RT	UltraView DAB Detection kit (Ventana Medical Systems, USA)		
CD163	MRQ-26	5973929001	Cell Marque, USA			Ready to use dispenser 32' 37°C			
Double staining CD68/CD163	514H12 / 10D6	MCA1815 / NCL-CD163	Bio-Rad, UK / Novocastra Germany	Whole mount sections.	10' boiling Tris/EDTA buffer at pH 9.0	1:50 / 1:100 Overnight, RT	Secondary antibodies: Goat-anti-Mouse IgG2a Alexa-Fluor-594 / Goat-anti-Mouse IgG1 Alexa-Fluor-488 (both from Life Technologies, USA)		DAPI

NCI, Netherlands Cancer Institute; RT, Room temperature; ‘, minutes; °C, degrees Celsius.
