# Supplementary material for: The Prognostic Value of Immune Factors in the Tumor Microenvironment of Penile Squamous Cell Carcinoma
Source: Front Immunol. 2018 Jun 11;9:1253. doi: 10.3389/fimmu.2018.01253 (PMC6004546; doi:10.3389/fimmu.2018.01253)
Supplement: Supplementary file 2 [file table_2.PDF]

# Supplementary Table 2: Tumor-microenvironmental characteristics.

| Variable | hrHPV$^-$ N=158 (%) | hrHPV$^+$ N=52 (%) | Total N=213 (%)^ | p-value* |
|---|---|---|---|---|
| **Total classical HLA expression** | | | | 0.36 |
| Normal | 17# (10.8) | 4 (7.7) | 21 (9.9) | |
| Partial downregulation | 40 (25.3) | 21 (40.4) | 62 (29.1) | |
| Complete downregulation | 25 (15.8) | 14 (26.9) | 39 (18.3) | |
| Missing | 76 (48.1) | 13 (25.0) | 91 (42.7) | |
| **Non-classical HLA score** | | | | 0.74 |
| Normal (absent) | 76 (48.1) | 32 (61.5) | 108 (50.7) | |
| Upregulated | 27 (17.1) | 13 (25.0) | 40 (18.8) | |
| Missing | 55 (34.8) | 7 (13.5) | 65 (30.5) | |
| **Tumour PD-L1** | | | | |
| Negative | 70 (44.3) | 32 (61.5) | 104 (48.8) | **0.03** (np) |
| Positive | 78 (49.4) | 17 (32.7) | 96 (45.1) | 0.09 (pat) |
| Diffuse | 29 (18.4) | 7 (13.5) | | |
| Margin | 49 (31.0) | 10 (19.2) | | |
| Missing | 10 (6.3) | 3 (5.8) | 13 (6.1) | |
| **Stromal PD-L1** | | | | 0.14 |
| Negative | 32 (20.3) | 15 (28.8) | 48 (22.5) | |
| Positive | 114 (72.2) | 34 (65.4) | 150 (70.4) | |
| Missing | 12 (7.6) | 3 (5.8) | 15 (7.0) | |
| **PD-L1+ TIM** | | | | 0.19 |
| Present | 44 (27.8) | 10 (19.2) | 140 (65.7) | |
| Absent | 99 (62.7) | 38 (73.1) | 54 (25.4) | |
| Missing | 15 (9.5) | 4 (4.4) | 19 (8.9) | |
| **Cytotoxic T-cells, CD8** | | | | |
| Intratumoral (median, (IQR)) | 13128.8 (19980) | 12209.5 (20287) | 12426.4 (19544) | 0.49 |
| Stromal (median, (IQR)) | 70798.2 (85803) | 60357.4 (80246) | 66633.0 (85510) | 0.09 |
| Missing (number, (%)) | 36 (22.8) | 2 (3.9) | 38 (17.8) | |
| **Regulatory T-cells, FoxP3** | | | | |
| Intratumoral (median, (IQR)) | 3638.8 (5715.8) | 3113.3 (3913.9) | 3463.3 (5250.6) | 0.16 |
| Stromal (median, (IQR)) | 25441.0 (26698.6) | 24907.4 (20230.7) | 25293.9 (25353.4) | 0.47 |
| Missing (number, (%)) | 11 (7.0) | 2 (3.9) | 14 (6.6) | |
| **T-cell ratio intratumoral** | | | | 0.29 |
| Median, (IQR) | 2.7529, (4.22) | 3.8101 (7.68) | 3.1074 (5.45) | |
| CD8>FoxP3 | 15 (9.5) | 6 (12.2) | 23 (10.8) | |
| CD8<FoxP3 | 95 (60.1) | 43 (81.7) | 138 (64.8) | |
| Missing | 45 (30.4) | 3 (5.8) | 52 (24.4) | |
| **T-cell ratio stromal** | | | | 0.33 |
| Median, IQR | 3.0899 (5.01) | 2.2455 (3.85) | 2.6186 (4.00) | |
| CD8>FoxP3 | 17 (10.8) | 6 (11.5) | 24 (11.3) | |
| CD8<FoxP3 | 83 (52.5) | 35 (67.3) | 119 (55.9) | |
| Missing | 58 (36.7) | 11 (21.2) | 70 (32.9) | |
| **Tumour macrophages, CD163** | | | | 0.28 |
| High tumour infiltration | 65 (41.1) | 29 (55.8) | 95 (44.6) | |

| | | | | |
|---|---|---|---|---|
| Low tumour infiltration | 89 (56.3) | 23 (44.2) | 114 (53.5) | |
| Missing | 4 (2.5) | - | 4 (1.9) | |
| **Stromal macrophages, CD163** | | | | 0.11 |
| High stromal infiltration | 123 (77.8) | 33 (63.5) | 157 (73.7) | |
| Low stromal infiltration | 30 (19.0) | 19 (36.5) | 51 (23.9) | |
| Missing | 5 (3.2) | - | 5 (2.3) | |

Np, as tested negative or positive; pat, as tested over the three patterns of expression (negative, diffuse or at tumour-stroma margin); IQR, interquartile range; SCC, squamous cell carcinoma; NOS, not otherwise specified, HPV, human papilloma virus; HLA, human leukocyte antigen; PD-L1, programmed death-ligand 1; TIM, tumour infiltrating macrophages.

^ Including three cases with unknown hrHPV status.

* Excluding missing cases. Comparing the two hrHPV-subgroups. Independent sample t-test for continuous variables, Chi-square or Fishers exact test for categorical variables.

# One case was excluded because the majority of other variables was missing.[5,7]
